# Entry, Retention, and Virological Suppression in an HIV Cohort Study in India: Description of the Cascade of Care and Implications for Reducing HIV-Related Mortality in Low- and Middle-Income Countries

**DOI:** 10.1155/2013/384805

**Published:** 2013-07-10

**Authors:** Gerardo Alvarez-Uria, Raghavakalyan Pakam, Manoranjan Midde, Praveen Kumar Naik

**Affiliations:** Department of Infectious Diseases, Rural Development Trust Hospital, Kadiri Road, Bathalapalli, Anantapur District, Andhra Pradesh 515661, India

## Abstract

HIV treatment, care, and support programmes in low- and middle-income countries have traditionally focused more on patients remaining in care after the initiation of antiretroviral therapy (ART) than on earlier stages of care. This study describes the cumulative retention from HIV diagnosis to the achievement of virological suppression after ART initiation in an HIV cohort study in India. Of all patients diagnosed with HIV, 70% entered into care within three months. 65% of patients ineligible for ART at the first assessment were retained in pre-ART care. 67% of those eligible for ART initiated treatment within three months. 30% of patients who initiated ART died or were lost to followup, and 82% achieved virological suppression in the last viral load determination. Most attrition occurred the in pre-ART stages of care, and it was estimated that only 31% of patients diagnosed with HIV engaged in care and achieved virological suppression after ART initiation. The total mortality attributable to pre-ART attrition was considerably higher than the mortality for not achieving virological suppression. This study indicates that early entry into pre-ART care along with timely initiation of ART is more likely to reduce HIV-related mortality compared to achieving virological suppression.

## 1. Introduction

By the end of 2011, more than 90% of the 34 million people infected with HIV worldwide were living in low- or middle-income countries [1]. 16.8$ billion was invested globally into HIV in 2011 and, in low- and middle-income countries, 89% of the investment was allocated to treatment care and support of HIV infected people [1]. 

One of the most important aims of the medical care of HIV patients is to initiate antiretroviral therapy (ART) before the development of HIV-related complications and to allow immunological recovery by maintaining long-term virological suppression. Despite important advances in the rollout of ART worldwide, 1.7 million people died of HIV-related pathologies in 2011 [1]. This high mortality could be explained by many factors including late presentation of HIV [2–5], poor engagement in medical care [6–9], poor retention in pre-ART care [10–12], late or no initiation of ART [13–15], high levels of attrition from care after ART initiation [16, 17], and poor virological suppression in patients on ART [18]. When designing programmes aimed at providing treatment, care, and support to people living with HIV, it is important to understand the contributions of these factors to the overall HIV mortality, in order to improve the efficiency of HIV spending. To date, the majority of HIV research and funding have focused on the reduction of mortality and morbidity after ART initiation. However, research also suggests that inadequate engagement in pre-ART care may result in fewer people receiving ART and may increase the proportion of patients initiating ART late [19]. 

Most research about the retention in HIV programmes comes from the United States and Sub-Saharan Africa [17, 19–21]. Despite 2.4 million HIV infected people estimated to be living with HIV in India in 2009 [22], only 486,173 were receiving ART under the national programme in January 2012 [23]. This study describes the engagement and retention in care of a large cohort of patients in Anantapur, India. In particular, we studied the proportion of patients who entered and were retained in care from the point of HIV diagnosis to HIV virological suppression. We aimed to estimate what stages were more important for reducing the mortality of people living with HIV.

## 2. Methods

### 2.1. Setting

The study was performed in Anantapur, a district situated in the south border of Andhra Pradesh, India, with a high prevalence of HIV infection in antenatal clinics [24]. The HIV epidemic in Anantapur is largely driven by heterosexual transmission and is characterized by poor socio-economic conditions and high levels of illiteracy in the HIV population [25]. Rural Development Trust (RDT) is a nongovernmental organization that provides medical care to HIV infected people free of cost, including medicines, consultations, hospital admission charges, CD4 cell count enumeration, and HIV viral load. The Vicente Ferrer HIV Cohort Study (VFHCS) is an open cohort study of all HIV infected patients who have attended RDT hospitals since June 2006. The characteristics of the cohort have been described in detail elsewhere [25]. For this study, we selected HIV infected adults (>15 years) living in Anantapur and diagnosed with HIV between January 1st 2007 and November 4th 2011. The selection of patients from the database was executed on September 14th 2012.

During this period, ART was available free of cost in the district, and the CD4 cell count threshold for initiating ART was 250 cells/*μ*L in accordance with the 2006 World Health Organization (WHO) guidelines [26–28]. Patients who were lost to followup (LTFU) were actively searched by phone calls and home visits by outreach workers, and in those patients who had died, relatives were asked about the date of death of the patient.

### 2.2. Definitions

To assess the HIV care before ART initiation, we divided the pre-ART care into three stages according to recent recommendations based on the experience of studies performed in Sub-Saharan Africa [19, 29]. To describe the entry into pre-ART care, we calculated the time between the HIV diagnosis and the first determination of the CD4 count (Stage 1). For HIV infected patients who were not eligible for ART at the first assessment, the Indian National Guidelines recommend performing CD4 count determinations every six months [26]. To describe the retention in pre-ART care of these patients (Stage 2), we calculated the number of completed semesters that patients had a CD4 count determination during the period starting at three months after the first CD4 count determination and finishing at the first CD4 cell count determination <250 cells/*μ*L, death, or November 4th 2011, whatever occurred first (follow-up period). To be considered retained in pre-ART care, patients should have had a CD4 count determination in all the semesters of followup. However, if a patient had a CD4 count >250 cells/*μ*L in the last 12 months of followup, they were considered retained in pre-ART care regardless of the number of previous semesters without CD4 count determinations [29]. To be included in the Stage 2 analysis, patients should have had at least a follow-up period of six months (one complete semester after three months from the first CD4 count determination). For example, for a patient who had the first CD4 count determination on October 1st, 2009 and died on January 15th, 2011, we checked whether the patient had a CD4 count determination from January 1st, 2010 (three months after the first CD4 count determination) to June 30th, 2010 (first semester) and from July 1st, 2010 to December 31th, 2010 (second semester). If he had CD4 count determinations during the first and second semesters, the patient was considered retained in care. If he had a CD4 count determination only in one semester, he did not meet criteria for being considered retained in pre-ART care. However, if the same patient had a CD4 lymphocyte count >250 cells/*μ*L in the last 12 months of followup, he was considered retained in care even if he did not have CD4 count determinations in all the semesters of followup. To describe the promptness of ART initiation, we calculated the time from the first CD4 count <250 cells/*μ*L to the initiation of ART (Stage 3). 

After ART initiation, patients were considered LTFU if they did not come to the clinics for at least 180 days after their last scheduled appointment. Patients on ART were considered retained in care if they did not stop ART for any reason, were not LTFU, and did not die (Stage 4). Virological suppression was defined as having an HIV viral load <400 copies/mL after six months of ART initiation in the last viral load determination (Stage 5). After completion of each stage, patients who transferred to other clinics were not included in the analysis of the next stage [29].

### 2.3. Statistical Analysis

Statistical analysis was performed using Stata Statistical software (Stata Corporation, Release 11, College Station, TX, USA). Confidence intervals for proportions were calculated using the Wilson method [30]. We used Kaplan-Meier survival estimates for calculating cumulative incidences of mortality for each stage of care. This study was approved by the Ethical Committee of the RDT Hospital.

## 3. Results

**Figure 1:**
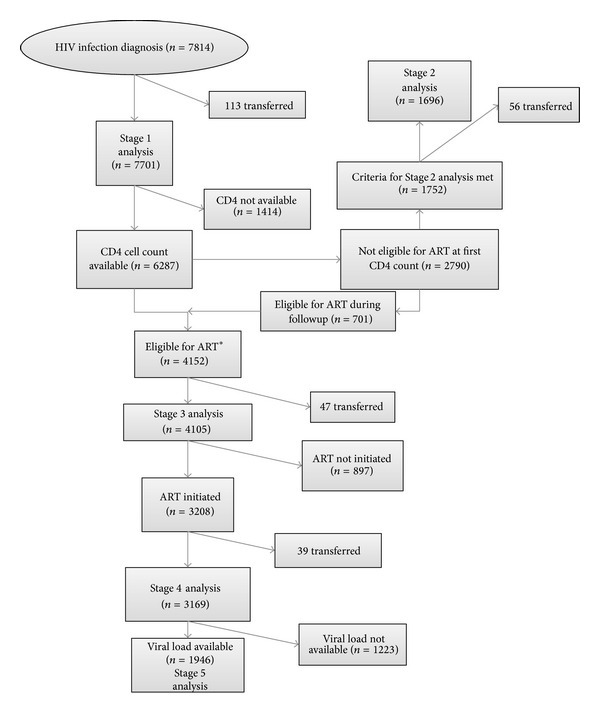
Flowchart of patients included in each stage of HIV care. ART, antiretroviral therapy; ∗ 46 patients having a first CD4 cell count <250 cells/*μ*L were excluded because the CD4 count determination was performed after November 4th 2011.

**Figure 2:**
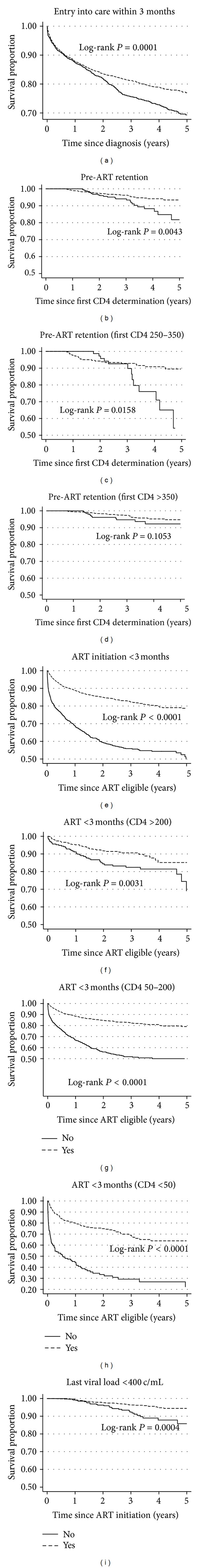
Survival curves at different stages of HIV care. Kaplan-Meier survival curves by entry into care within 3 months (a), by retention in pre-ART care (b), by retention in pre-ART care of patients with initial CD4 count 250–350 (c), >350 (d), by ART initiation within 3 months of becoming eligible (e), by ART initiation within three months of patients with CD4 count >200 (f), 50–200 (g), <50 (h), and by virological suppression in the last viral load determination (i). ART, antiretroviral therapy; CD4, CD4 count in cells/*μ*L; c/mL, copies/mL.

The number of patients included in each stage is summarized in Figure 1. We identified 7814 patients diagnosed with HIV infection and 7701 were included in the Stage 1 analysis. 6287 (81.6%) patients had a CD4 count determination. Of 2790 patients who were not eligible for ART at the first assessment, 1696 patients met the inclusion criteria for the Stage 2 analysis. During the study period, we identified 4152 patients who were eligible for ART initiation, and 4105 of them were included in the Stage 3 analysis. ART was initiated in 3208 (78.1%) patients and 3169 were included in the Stage 4 analysis. Of these 3169 patients, 1946 (61.4%) had a viral load determination after six months of ART and they were included in the Stage 5 analysis. Kaplan-Meier survival curves at different stages of HIV care are presented in Figure 2.

### 3.1. Stage 1

Of 7701 patients diagnosed with HIV infection, 70.3% (*n* = 5410) entered into care within three months of HIV diagnosis. The median CD4 count of patients who entered into care was 216 cells/*μ*L (interquartile range [IQR], 106–398). Kaplan-Meier survival curves at Stage 1 are presented in Figure 2(a). Patients who entered into care within three months of HIV diagnosis had lower mortality than patients who did not (*P* = 0.0001). The estimated five-year mortality difference between the two groups was 7.5% (95% CI, 4.2–10.9).

### 3.2. Stage 2

Of 1696 patients included in the Stage 2 analysis, 26.3% (*n* = 446) had CD4 count determinations in all semesters of followup. Of 1250 patients who did not have CD4 count determinations in all semesters, 662 (53%) had a CD4 count >250 cells/*μ*L in the last 12 months of followup. Hence, 65.3% (*n* = 1108) were considered as retained in pre-ART care according to study criteria. The median pre-ART followup was three semesters (IQR, 1–5). Kaplan-Meier survival curves at Stage 2 are presented in Figure 2(b). Patients who were retained in pre-ART care had lower mortality than patients who were not retained in pre-ART care (*P* = 0.0043). The estimated five-year mortality difference between the two groups was 11.7% (95% CI, 2.7–20.6). However, when comparing mortality by CD4 count at entry into care (Figures 2(c) and 2(d)), we did not observe significant differences in patients having CD4 count >350 cells/*μ*L (*P* = 0.1053). 

### 3.3. Stage 3

Of 4105 patients eligible for ART, 67.3% (*n* = 2761) initiated ART within three months of becoming eligible. Kaplan-Meier survival curves at Stage 3 are presented in Figure 2(e). Patients who initiated ART within three months had lower mortality than patients who did not (*P* < 0.0001). The estimated five-year mortality difference between the two groups was 28.7% (95% CI, 23–34.4). Differences in mortality were more pronounced for patients with CD4 count <200 cells/*μ*L (Figures 2(f), 2(g), and 2(h)). Looking at the Kaplan-Meier survival curves for Stage 3, we can observe that an important proportion of patients who did not initiate ART within three months died during the initial period of followup. Considering that many of these patients might be too sick to initiate ART and that the positive effect of ART on survival is observed only after a few weeks of treatment [31], we also calculated the five-year mortality differences between patients who did and did not initiate ART within three months including only those patients having >3 months of followup (i.e., did not die or were not LTFU within three months of becoming eligible for ART), which was 19.9% (95% CI, 13–26.7).

### 3.4. Stages 4 and 5

Of 3169 patients who initiated ART, 15% (*n* = 480) died, 15% (*n* = 474) were LTFU, and 0.7% (*n* = 23) stopped ART. Of 1946 patients who had a viral load determination after six months of ART initiation, 81.8% (*n* = 1591) achieved virological suppression (<400 copies/mL) in the last viral load determination. The median time from the ART initiation to the last viral load determination was 2.26 years (IQR, 1.33–3.31). Kaplan-Meier survival curves at Stage 5 are presented in Figure 2(i). Patients who achieved virological suppression had lower mortality than patients who did not achieve virological suppression (*P* = 0.0004). The estimated five-year mortality difference between the two groups was 8.7% (95% CI, 2.3–15).

### 3.5. Combinations of All Stages

**Figure 3:**
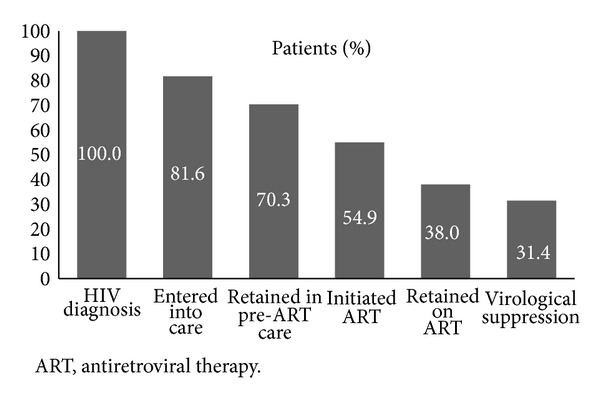
Cumulative retention of patients at each stage of care after HIV diagnosis.

Outcomes for each stage of HIV care are summarized in detail in Table 1. Of 7701 patients diagnosed with HIV, 1414 (18.4%) did not enter into care. Of 1696 patients who were ART ineligible at the first assessment and met inclusion criteria for the Stage 2 analysis, 531 (31.3%) were not retained in pre-ART care and did not come back to the clinics, so we estimated that 31.3% of the 44.4% of patients who were not eligible for ART at the first assessment (i.e., 13.9% of all patients who entered into care) were not retained in pre-ART care. Of 4105 patients who were eligible for ART, 897 (21.9%) did not initiate ART. Of 3169 patients who initiated ART, 977 (30.8%) were not retained in care. Of 1790 patients who were retained in care after ART initiation (i.e., did not die, were not LTFU, and did not stop ART) and had a viral load determination after six months of initiating ART, 309 (17.3%) did not achieve virological control (<400 copies/mL) in the last viral load determination. Taking into account the attrition at each stage, we calculated the cumulative retention in care during the period of the study (Figure 3). According to this estimation, only 31.4% of patients diagnosed with HIV in our setting followed all stages of care.

**Figure 4:**
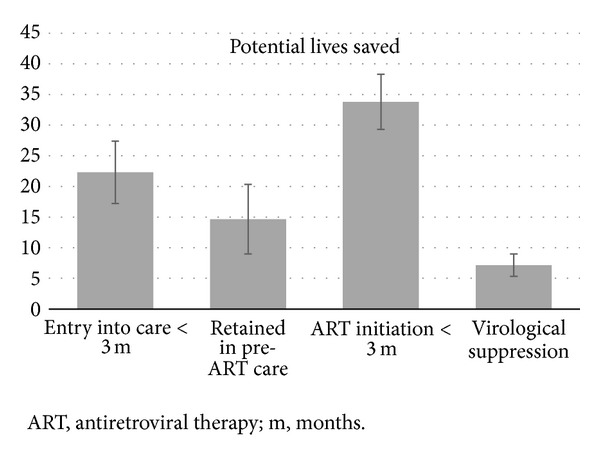
Potential five-year mortality reduction of 1000 patients diagnosed with HIV infection (error  bars = standard  errors).

In Figure 4, we made a simulation of the number of potential lives saved out of 1000 patients diagnosed with HIV with hypothetical HIV programmes that could achieve a 100% success in the different stages of HIV care. The calculations were performed taking into account: (i) the differences in the five-year mortality between patients who did and did not achieve the objectives for Stage 1 (entry into care within three months), Stage 2 (retention in pre-ART care prior to ART eligibility), Stage 3 (initiation of ART within three months of eligibility), and Stage 5 (virological suppression); (ii) the cumulative retention for Stage 1 (patients with HIV diagnosis, i.e., 100%), Stage 2 (patients who entered into care but were ineligible for ART at the first assessment, i.e., 36.2%), Stage 3 (patients retained in pre-ART care, i.e., 70.3%), and Stage 5 (patients retained in care >6 months after ART initiation, i.e., 45.4%); (iii) the proportion of patients who did not achieve the objectives for Stages 1, 2, 3, and 5 (Table 1). Some patients who died within three months of ART eligibility did not initiate ART because they were too sick and early initiation of ART would not have improved their prognosis [31]. Thus, with the intention of being conservative in our calculations, we used the five-year mortality difference between patients who did and did not initiate ART within three months of ART eligibility who had >3 months of followup to estimate the potential impact of improving Stage 3 in HIV programmes. Nevertheless, the highest potential impact for reducing the mortality in HIV programmes was observed at Stage 3 (3.38%, 95% CI 2.21–4.54) followed by Stage 1 (2.23%, 95% CI 1.24–3.23), Stage 2 (1.47%, 95% CI 0.34–2.59), and Stage 5 (0.72%, 95% CI 0.19–1.24).

## 4. Discussion 

In this cohort study, we found that less than one-third of people diagnosed with HIV followed all stages of care from diagnosis to the achievement of virological suppression after ART initiation, and that attrition was higher in pre-ART stages than after ART initiation. Although the study is limited to a small area of India and comparisons among studies are difficult because of different operational definitions [29], our results are in accordance with other studies performed in resource-limited settings. In two systematic reviews of the retention in care in Sub-Saharan Africa, less than one third of patients were retained in care prior to ART initiation, and only 65%, of those who initiated ART, were retained in care after three years of followup [17, 19]. In a meta-analysis of the pre-ART attrition in Sub-Saharan Africa [21], the attrition in Stage 1 was 22.4% and the attrition in Stage 3 was 37.1%. In a single site cohort study in South Africa [32], the cumulative retention in care of ART eligible patients from HIV diagnosis to 6–12 months after ART initiation was 36.9%. Although these studies differ in some of the stage definitions and follow-up periods, the rates of attrition are similar to our study, suggesting comparable rates of attrition in Sub-Saharan Africa and India. 

Despite notable achievements, the mortality due to HIV in developing countries is still high [1]. Governments in low- and middle-income countries are making efforts to reduce this high mortality, but resources and monitoring are focused more on patients on ART than on pre-ART stages of care [33]. However, the results of this study show that improving pre-ART stages of care can have greater impact on reducing HIV mortality than achieving virological suppression. Moreover, previous studies have shown that interventions to improve engagement in care can be cost-effective [34–36]. These data indicate that stakeholders and donors should consider placing more emphasis on promoting research and implementing evidence-based interventions to improve entry and retention in pre-ART care [37, 38].

To our knowledge, this is one of the first studies to follow patients from the diagnosis of HIV to the achievement of virological suppression in a resource-limited setting. In the United States, it is estimated that 80% of HIV infected patients have been diagnosed with HIV; 77% of patients diagnosed with HIV enter into care within 3-4 months of diagnosis; 66.2% of patients linked to care are retained in care; and 77% of patients on ART achieve virological suppression [20]. In our cohort, 70.3% of patients diagnosed with HIV entered into care within three months; 46.5% of patients linked to care were retained in care; and 81.8% of patients on ART achieved virological suppression. The total number of people diagnosed with HIV in the district is not known, but in 2011 it is estimated that the number of people living with HIV was 26,800, and by November 2011, 14,395 patients were registered in Government ART Centres [22, 39–41]. Therefore, only 54% of the estimated HIV infected people in the district entered into care, which compares unfavourably with the 61.6% HIV infected patients who enter into care in the United States [20]. A similar observation is made with the difference in CD4 counts at entry into care in our cohort (216 cells/*μ*L, IQR 106–398) compared with the United States and Canada (317 cells/*μ*L, IQR 135–517) [2]. These data suggest that, compared to North America, in our setting a lower proportion of patients enter into care, initial CD4 counts are lower, there is more attrition (mortality and loss to follow up) after engagement in care, and similar proportions of patients achieve virological suppression. 

In our study, there was no significant increase in mortality among patients who had an initial CD4 count >350 cells/*μ*L and poor retention in pre-ART care. In the 2010 WHO guidelines [33], the CD4 count threshold for initiating ART has been raised to 350 cells/*μ*L. Thus, the proportion of patients who will become eligible for ART soon after the HIV diagnosis will increase considerably [5]. Previous studies have shown a reduction in mortality and loss to follow up when ART is initiated with CD4 >200 cells/*μ*L [42–44]. Therefore, it is likely that the implementation of the 2010 WHO guidelines will reduce both the mortality due to poor retention in pre-ART care and attrition after ART initiation. 

To define the stages of pre-ART care, we used the date of collection of the CD4 count sample, rather than the date patients are informed of their CD4 count result. Although the CD4 count notification date has been recommended by some authors [29], most patients are informed of their results at the next clinical visit, which for patients in pre-ART care may occur many months later [11, 32]. Using the CD4 count sample date to monitor the performance of HIV programmes in pre-ART stages can encourage these programmes to implement effective strategies (e.g., phone calls, home visits, etc.) aimed at informing ART eligible patients of the need for prompt initiation of therapy.

The study has some limitations. The proportion of patients diagnosed with HIV who never entered into care is likely to be underestimated. HIV testing is performed in different locations, and patients diagnosed in other healthcare facilities and never entered into care were not identified. We estimated the proportion of ART-ineligible patients who were not retained in pre-ART care, but not all of them were followed up until becoming eligible for ART. Therefore, it is possible that longer follow-up periods may result in higher rates of Stage 2 attrition. Moreover, patients LTFU at any stage may not be lost forever, as they may reengage in the future or enroll in other ART centres. However, patients LTFU are more likely to initiate ART with low CD4 counts or may die before attending other healthcare facilities [45]. 

## 5. Conclusions

This study shows that less than one third of patients diagnosed with HIV in our setting follow all the stages of care up to the achievement of virological suppression after ART initiation. Most attrition occurs in pre-ART stages, and the mortality attributable to pre-ART attrition is considerably higher than the mortality due to lack of virological suppression after ART initiation. Considering that other studies in low- and middle-income countries had similar results, it should be recommended that HIV programmes monitor their performance in pre-ART stages of care. Implementing new strategies to improve the link between HIV testing and pre-ART care and timely initiation of ART would help reduce the mortality of people living with HIV in resource-limited settings.

## Figures and Tables

**Table 1:** Outcomes at each stage of HIV care.

	% (95% CI)
Entry into care (*n* = 7701)	
<3 months	70.3 (69.2–71.3)
3–6 months	4.5 (4–5)
6–9 months	2 (1.7–2.4)
9–12 months	1.1 (0.9–1.4)
>12 months	3.8 (3.4–4.2)
No entry	18.4 (17.5–19.2)
Retention in pre-ART care (*n* = 1696)	
100%	26.3 (24.3–28.4)
75–99.9%	9 (7.7–10.5)
50–74.9%	23 (21.1–25.1)
<50%	12.7 (11.2–14.4)
0%	29 (26.8–31.2)
CD4 > 250 in the last year of followup	56 (53.6–58.3)
ART initiation (*n* = 4105)	
<3 months	67.3 (65.8–68.7)
3–6 months	4.6 (4–5.2)
6–9 months	2.3 (1.9–2.8)
9–12 months	0.9 (0.7–1.3)
>12 months	3.1 (2.6–3.6)
No ART initiation	21.9 (20.6–23.1)
Retention in ART care (*n* = 3169)	
Died	15.1 (13.9–16.4)
ART stopped	0.7 (0.5–1.1)
LTFU	15 (13.8–16.2)
Alive and on ART	69.2 (67.5–70.8)
Last viral load <400 c/mL (*n* = 1946)	
No	18.2 (16.6–20)
Yes	81.8 (80–83.4)

ART: antiretroviral therapy; CI: confidence interval; LTFU: lost to followup.
